# Influencing factors of patients’ behavior of healthcare seeking: a meta-analysis in China

**DOI:** 10.3389/fpubh.2025.1583075

**Published:** 2025-04-28

**Authors:** Yongtao Song, Mingzhe Wu, Hailong Feng

**Affiliations:** ^1^School of Business, Henan University, Kaifeng, China; ^2^School of Business Administration, Anhui University of Finance and Economics, Bengbu, China

**Keywords:** behavior of healthcare seeking, meta-analysis, influencing factors, hierarchical medical system, moderating effect analysis

## Abstract

**Objectives:**

Understanding patient’s healthcare seeking behavior (BHS) and identifying the determinants influencing BHS can optimize the allocation of medical resources and enhance the efficacy of healthcare systems. The purpose of this study is to identify the influencing factors of patients’ BHS and to assess the extent and variation in their impact on BHS.

**Methods:**

Drawing upon the Anderson Behavioral Model of Health Service Utilization, we summarized the factors influencing patients’ BHS into three categories, and examined empirical journal articles published from 2010 to 2023 using meta-analysis. In addition, the Bayesian analysis of variance was introduced to explore the influence of moderators.

**Results:**

A total of 39 empirical journal articles were finally identified for meta-analysis. Ten out of the thirteen factors have a significant and positive influence on BHS, with the exception of trust in medical institutions (*z* = 0.176, *p* = 0.077), health record (*z* = 1.942, *p* = 0.052), and medical expenses (*z* = 0.1846, *p* = 0.065). The results of moderating effect analysis indicate that there is a significant difference in the impact of age (*p* < 0.001), education level (*p* < 0.05), family income (*p* < 0.001), medical insurance (*p* < 0.001), illness severity (*p* < 0.01), and healthcare service reputation (*p* < 0.001) on BHS.

**Conclusion:**

There is a positive and significant influence of the antecedent variables (self-rated health, distance to medical facilities, illness severity, age, family income, education level, marital status, medical insurance, awareness of hierarchical healthcare, health record, and healthcare service reputation) on BHS. Furthermore, the influences of family income, medical insurance, and illness severity on BHS in developed areas are stronger than that in underdeveloped areas, while the influences of age, education level, and healthcare service reputation on BHS in underdeveloped areas are stronger than that in developed areas.

## Highlights

- We summarize the factors influencing Chinese patients’ behavior of healthcare seeking into demographic factors, individual cognitive factors and medical-level factors, and analyze their influences on patients’ behavior of healthcare seeking.
- Ten out of the thirteen antecedent factors have a positive influence on patients’ behavior of healthcare seeking, and the influence of these factors is moderated by regional economic development level.
- The study makes theoretical contributions to the design of healthcare service systems, especially in the research on patients’ behavior of healthcare seeking in developing countries.

## 1 Introduction

Hierarchical medical system is a key constituent of the healthcare service systems in most countries. The main feature of these healthcare service systems is that medical institutions are distributed in a pyramid shape, and patients must go through primary healthcare institutions to enter the national healthcare service system (1). The purpose of hierarchical medical system is to provide basic health services at the lowest cost in the face of a shortage of medical resources (2), and to protect specialists’ resources (3). To address the shortage of high-quality medical resources and relieve the long-standing problem of difficulty of receiving medical treatment, China has planned a three-level healthcare system that covers primary healthcare institutions, second-class hospitals, and tertiary hospitals (4).

In the planned hierarchical medical system, primary healthcare institutions are responsible for providing treatment for patients with clear diagnoses and stable conditions and rehabilitation nursing services for chronic disease patients, while tertiary hospitals are responsible for providing diagnosis and treatment services for severe and complicated diseases. First visiting patients need to visit the primary healthcare institutions firstly before they move up to the more sophisticated institutions for follow-up treatment (2). The primary healthcare institutions decide whether to treat patients or refer them to higher-level hospitals based on diagnostic results (3). In such a hierarchical medical system, primary healthcare institutions serve as a gatekeeper for the healthcare system with the aim to maximize the value of medical resources. However, in China, patients do not actually behave as the hierarchical medical system expects them to and often visit tertiary hospitals first regardless of the severity of illness (5). This situation leads to overcrowding and a serious lack of reception capacity in tertiary hospitals, while the medical resources of primary hospitals are idle (6). Primary care has not served as a gatekeeper for the healthcare system in China. Mwabu (2) proposed that the effectiveness of hierarchical medical system depends on how well it reflects the patient’s behavior of healthcare-seeking. With this issue in mind, it is necessary to examine and understand Chinese patients’ BHS and to identify the important factors that affect patients’ BHS.

Academics and those responsible for public medical policy have steered their efforts toward identifying the influencing factors of patients’ BHS, such as individual characteristics (7, 8), patient trust (9), quality of medical services (10, 11), medical expenses (12), accessibility of medical services (12, 13), and convenience of seeking medical treatment (14). Despite these significant efforts, some relevant gaps still need to be addressed. Firstly, the results of different studies are inconsistent and even contradictory. For example, some studies found that age, education level, and family income significantly affect patients’ BHS (15, 16), while other studies found that the influence of above factors on patients’ BHS is insignificant (17). Stricter quantitative techniques are needed to integrate current inconsistent research conclusions (18). Secondly, existing researches focused on different factors influencing BHS, which is not conducive to a systematic understanding of patients’ BHS and guiding practice.

In this paper, we address the following questions: (1) What are the factors that influence patients’ BHS in China? (2) What are the boundary conditions that various factors influence patients’ BHS? This study uses meta-analysis to analyze the factors influencing Chinese patients’ BHS. Meta-analysis is a research method that systematically integrates multiple quantitative research results in a research field, and it can correct the statistical artefacts in existing studies and provide a better aggregate estimation (19). Furthermore, meta-analysis is conducive to recognizing trends that cannot be observed in individual studies and producing results that are more meaningful than those of individual studies (20). When there exists contradictor or fragmented conclusions, meta-analysis can explore the contingency characteristics of variable relationships and discover new research pathways by integrating studies from different backgrounds (21).

The rest of the paper is organized as follows. The second part provides the theoretical background and relevant hypotheses of this article. The third part introduces the research methods and analysis process of this article. The fourth part presents the results of meta-analysis, and the fifth part discusses the conclusions, theoretical and practical implications, limitations, and suggests a number of future research directions.

## 2 Theoretical background and hypotheses

**Figure 1:**
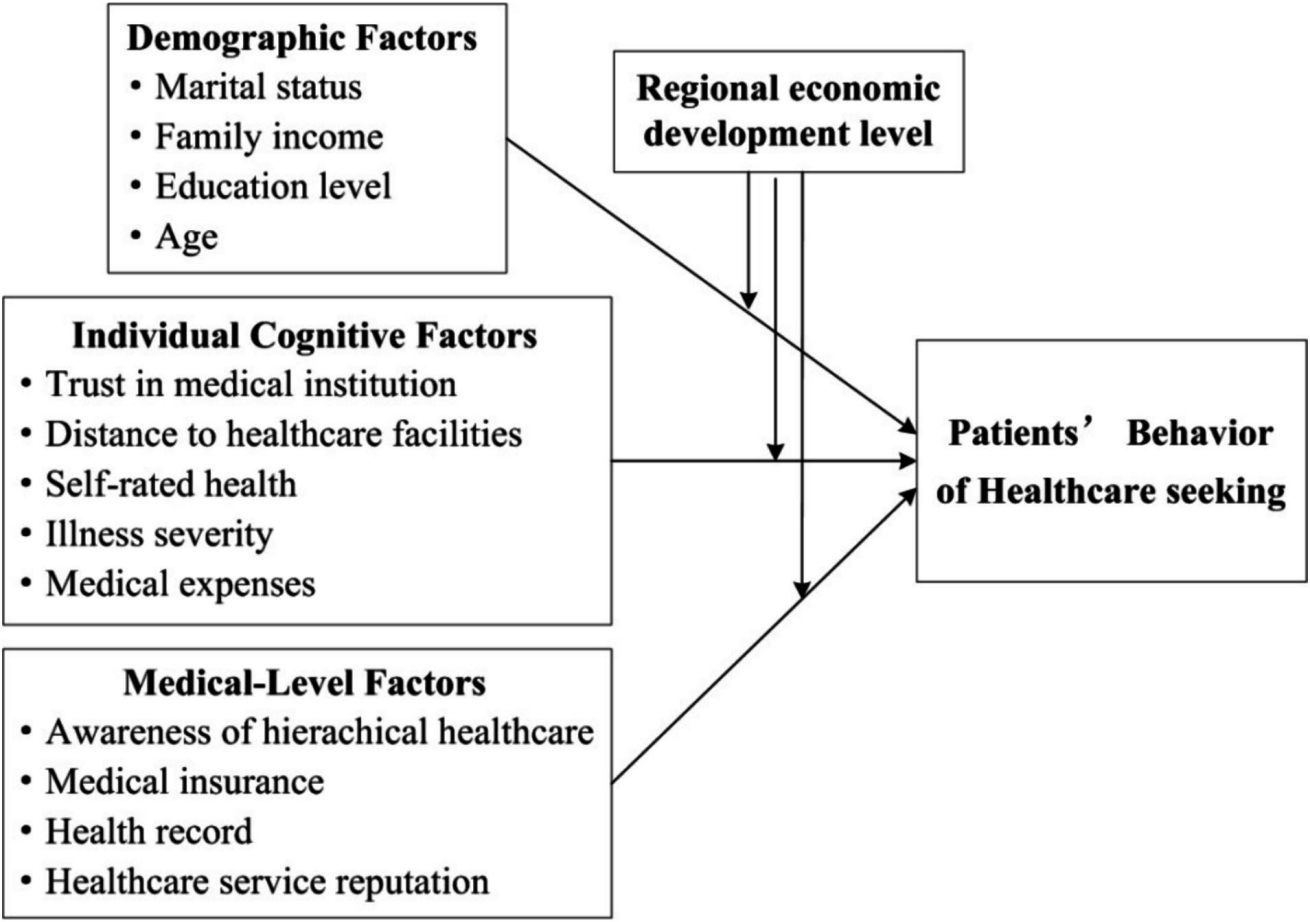
Research model.

The Anderson Behavior Model of Health Services Utilization (ABMHSU) stands out as a classic and influential theoretical framework extensively applied across public health, medicine, and sociology disciplines (22). ABMHSU proposes that patients’ BHS is a product of multifaceted interactions among various factors. These factors encompass not only individual characteristics such as age, gender, education, healthcare perceptions, and health status, but also the enabling resources available to individuals and their families, including economic status, medical insurance coverage, and transportation accessibility (23). According to ABMHSU, we summarize the factors influencing patients’ BHS into three categories: demographic factors, individual cognitive factors, and medical-level factors (Figure 1).

### 2.1 Demographic factors and patients’ BHS

Most studies have confirmed the influence of age and education level on BHS. Thompson et al. (24) found that young patients are more willing to seek medical advice compared to older adult patients for mental health issues. Through screening of tuberculosis patients, Thomas et al. (25) found that age has a significant effect on BHS of tuberculosis patients. In terms of educational level, through the analysis of 3,230 tuberculosis patients in five studies, Gamtesa et al. (26) found that education level is one of the main factors that predict tuberculosis patients to seek medical advice or not. An empirical study conducted by Kundu et al. (27) investigates the mortality rate and healthcare seeking situation for pneumonia in children under the age of five in Bangladesh from 2007 to 2017, and indicates that the mortality rate and healthcare seeking situation for pneumonia in children largely depend on the mother’s education level. Therefore, based on the viewpoints of existing research, we propose the following hypotheses:

*H1*: Age is positively related to patients’ BHS.

*H2*: Educational level is positively related to patients’ BHS.

Family income largely determines a patient’s financial capacity for healthcare services. Tesema and Seifu (16) conducted a survey on mothers of children under the 5 year old in sub-Saharan Africa and found that mothers from high-income families can afford high medical expenses, so they are able to actively seek medical attention when their children are sick. Similarly, Zewude et al. (28) investigated respondents in Ethiopia who had never visited doctors and found that the main factor influencing these people not visiting doctors is household income. In terms of marital status, Tekalign et al. (29) found that children with single mothers were 65% less likely to seek medical treatment than children with non-single mothers. Cheng et al. (7) conducted a survey on patients who visited primary healthcare institutions and found that single patients preferred to choose primary healthcare institutions nearby due to a lack of family companionship. Based on the viewpoints of existing research, we propose:

*H3*: Family income is positively related to patients’ BHS.

*H4*: Marital status is positively related to patients’ BHS.

### 2.2 Individual cognitive factors and patients’ BHS

Individual cognition plays a critical role in patients’ medical decision-making and BHS (30). Extensive studies have found that, due to the credence goods attribute of healthcare services (11), patients’ trust in hospitals and service providers stemmed from the reputation of healthcare services is the key to determining their BHS. Thompson et al. (24) conducted a survey of patients’ experiences and found that trust in hospitals is a key factor affecting patients’ BHS. Nab et al. (9) analyzed the characteristics of patients who delayed visiting doctor during the COVID-19 pandemic and found that the main factor leading to patients’ delayed medical treatment is low trust in medical institutions. Based on this, this study proposes:

*H5*: Trust in medical institution is positively related to patients’ BHS.

The purchasing cost of products/services is one of the key factors for consumers when making purchasing decisions. Healthcare is a typical service and its purchasing cost also is a key factor that patients will consider when making medical decisions. Pu et al. (31) investigated the influencing factors of patients’ BHS and found that high-level hospitals have high fees, which makes it difficult for low-income older adult people to receive timely treatment when suffering from serious illnesses. An investigation into residents’ Initial primary care visit under the hierarchical medical system indicates that patients will choose primary care when the expenses do not exceed 1,000 RMB, and patients tend to prefer general hospitals when the expenses exceed 1,000 RMB (32). Based on this, this study proposes:

*H6*: Medical expenses are positively related to patients’ BHS.

The convenience of healthcare service is also one of the key factors affecting patients’ BHS (14). As the main variable representing the convenience of healthcare service, the distance to healthcare facilities has been widely addressed in existing research. Sivey (33) investigated the impact of hospital distance and waiting time during healthcare delivery on patients’ choice of service provider for cataract patients, and found that distance from healthcare institutions had a greater impact on patients’ choice of service provider than waiting time during healthcare delivery. Using multivariate logistic regression, Kombate et al. (13) found that there is a significant correlation between BHS of patients under 5 years old and their distance to healthcare facilities. Likewise, Akter et al. (34) conducted an investigation into infectious diseases among children living in slums in Bangladesh, and found that respondents who lived closer to healthcare institutions (≤ 30 min’ walking distance) were more likely to visit doctor when they fell ill than those who lived further away. Based on the above research, we propose:

*H7*: The distance to healthcare facilities is positively related to patients’ BHS.

In terms of self-rated health, Zeng et al. (15) analyzed patients’ medical preferences and resource utilization of community healthcare service centers in Xiamen, and found that chronic disease patients with good status of self-rated health are more inclined to visit to community health service centers rather than tertiary hospitals. Another investigation into over 7,400 patients by Agarwal et al. (35) found that, patients with average or poor self-rated health were more inclined to seek medical attention. In terms of perception of illness severity, Handebo et al. (8) found that the illness severity of a child perceived by the mother is the main factor determining whether and how to visit a doctor, mothers perceived their child to have a serious illness are 4.04 times more likely to visit a doctor than mothers who perceive their child to have a low severity of illness. Likewise, a survey of 600 chronic disease patients in Xuzhou, China by Li et al., (5) found that patients tend to visit primary healthcare institutions when they perceive the disease to be mild, and tend to visit higher-level hospitals when they perceive the disease to be severe. Based on this, we propose:

*H8*: Self-rated health is positively related to patients’ BHS.

*H9*: Illness severity is positively related to patients’ BHS.

### 2.3 Medical-level factors and patients’ BHS

It is commonplace to assert that, as the main content of healthcare policies, the basic medical insurance plays an important role in optimizing the allocation of medical resources and guiding patients to seek healthcare service reasonably. Against the backdrop of Chinese healthcare system and medical insurance system, Jin et al. (36) investigated the influence of medical insurance reimbursement on patients’ BHS and found that, a higher medical insurance reimbursement ratio would guide patients to visit primary healthcare institution firstly. Further to this viewpoint, an empirical study by Cao et al. (37) indicated that whether residents participate in basic medical insurance has a significant impact on guiding them to visit primary healthcare institutions. Furthermore, due to the differences in the urban–rural dual structure, there are significant differences between the New Rural Cooperative Medical Scheme and the Urban Rural Resident Medical Insurance in guiding residents to visit primary healthcare institution (37). This view is shared by Wu et al. (38), who found patients participating in the Urban Rural Resident Medical Insurance have a lower probability of medical deviation behavior. Thus, we present the following hypothesis:

*H10*: Medical insurance is positively related to patients’ BHS.

Product/service quality is one of the main determining factors in consumer purchasing decisions. However, due to the credence goods attribute of healthcare service, patients cannot obtain authentic quality information of healthcare services. Therefore, patients’ BHS largely rely on external information, such as hospital reputation and patient evaluations. Wang et al. (39) found that patients are very concerned about the reputation of service provider when making medical decisions, and are more willing to share disease information and their own health information with doctors with good reputations. Qiu et al. (40) also found that patients tend to consider the reputation of hospitals as a key factor in choosing doctors. Based on this, we present the following hypothesis:

*H11*: Healthcare Service Reputation of healthcare institutions is positively related to patients’ BHS.

With the increasing awareness of health, the public not only pays attention to the treatment of diseases, but also to health management based on disease prevention. It is no wonder that whether healthcare institutions establish health record for patients and help them monitor their own health status in real time, and eventually provide continuous health guidance to patients, to a certain extent, affects patients’ BHS. A survey of chronic disease patients in Shaanxi province by Lai et al. (41) indicated that, health record increases residents’ awareness of self-care. In more concrete terms, chronic disease patients with health record are more inclined to seek healthcare service when they are ill (41). Thus, we present the following hypothesis:

*H12*: Health record is positively related to patients’ BHS.

Although China has planned a three-level healthcare system, it failed to effectively exert the function of guiding patients for primary diagnosis. In Zhang et al. (32) opinion, the proportion of the public who are not familiar with the hierarchical medical system visiting primary healthcare institutions when they are ill is relatively low. Therefore, public’s awareness of hierarchical medical system can increase their probability of visiting primary healthcare institutions. Especially under hierarchical medical system, patients with primary care experience are more inclined to choose primary healthcare institutions for their next illness due to their personal experience (42). Thus, we present the following hypothesis:

*H13*: Awareness of hierarchical healthcare is positively related to patients’ BHS.

## 3 Methodology

### 3.1 Literature search and screening

The purpose of this article is to identify the influencing factors of Chinese patients’ BHS and to examine the influence of these factors on patients’ BHS. We searched studies published in English and Chinese between January 2010 and December 2023. First, we searched journal articles and conference proceedings in the following databases: Web of Science, Science Direct, Wiley Online Library, EBSCO host, Emerald insight, China National Knowledge Infrastructure (CKNI), and China Science and Technology Journal Database (CSTJ). Second, we used various possible synonyms for behavior of healthcare seeking to ensure no relevant paper is omitted, including ‘healthcare seeking’ or ‘health seeking’ or ‘medical seeking’ or ‘medical’ and ‘behavior’ or ‘decision’ or “willingness,” ‘medical help seeking behavior’, ‘seek medical advice’, ‘hospital visiting’, and ‘doctor visiting’.

**Figure 2:**
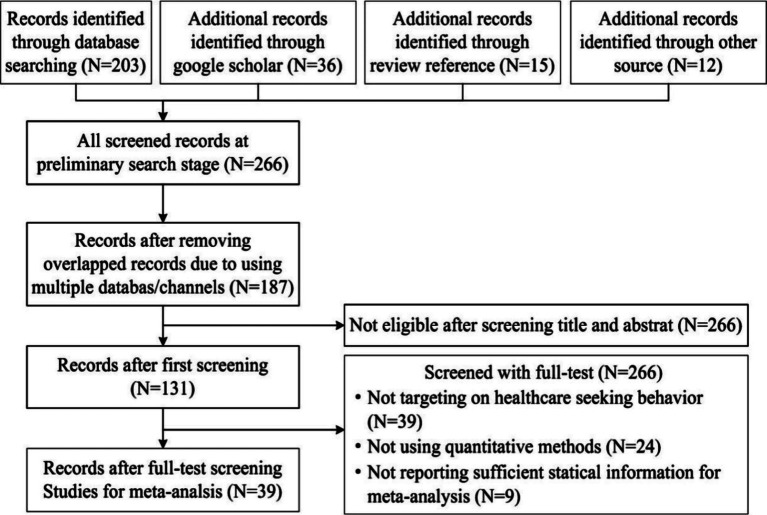
Flow chart of the study selection process.

The research process of this study is descripted in Figure 2. The initial search resulted in a total of 266 articles. These articles were screened using the following criteria in light of existing mate-analysis literatures: (1) relevant literature must be empirical research and exclude literature that uses qualitative research methods; (2) the literature must be independent studies that do not contain the same sample; (3) the dependent variable of the literature must be BHS (behavior of medical seeking, healthcare decision, willingness to seek healthcare, etc.); (4) the sample used in the study must be from China; (5) the correlation coefficients and sample size, or correlation coefficients and standard error, or correlation coefficient and the value of *p* must be reported (19). Based on above-mentioned criteria, 39 articles were finally identified for meta-analysis (Table 1). These 39 articles contain 39 independent samples, with a total sample size of 168, 289 and an average sample size of 4, 315.

**Table 1:** Original literature included in the meta-analysis.

Author	Subject area	Sample size	Influence factor	outcome
Liu et al. (54)	Shaanxi, China	450	EDL, AGE, FAI, AHH, TMI	Behavior of health care seeking
Chen et al. (55)	Northeast, west China	1,340	MEI, SRH, MAS, AGE	Behavior of health care seeking
Lai et al. (41)	Shaanxi, China	8,784	FAI, HER, ILS, AGE, DTM, SRH	Behavior of health care seeking
Wang et al. (56)	Rural China	9,712	MAS, ILS, MEI, MEE, AGE, EDL, FAI	Behavior of health care seeking
Zhang et al. (57)	Central Region of China	16,101	ILS	Medical Treatment Choice
Song et al. (58)	Northeast, west China	3,388	AGE, SRH, FAI, MEI, HER, MAS	Behavior of health care seeking
Zeng et al. (59)	Economically developed regions	12,938	MSR	Patients’ Behavior of Selecting Physicians
Wu et al. (60)	Underdeveloped regions	229	MEE, MSR	Preferences for doctor selection
Yi et al. (61)	Unspecified area	8,053	MSR	Patients’ Behavior of Selecting Physicians
Wei et al. (62)	Shanghai, China	6,659	MSR	Patients’ Behavior of Selecting Doctors
Wu et al. (63)	Economically developed regions	18,335	MSR	Selection of a Physician by Patients
Li et al. (64)	Shanghai, China	9,797	AGE, FAI, MAS	Behavior of health care seeking
Feng et al. (65)	Underdeveloped regions	2077	EDL, MEI, TMI, HER, DTM	Behavior of health care seeking
He et al. (66)	Western, Eastern, and Central regions of China.	5,657	EDL, HER, MEI, DTM, SRH	Behavior of health care seeking
Li et al. (67)	Jiangsu, China	2,498	MEI	Behavior of health care seeking
He et al. (68)	Beijing, China	355	AGE, FAI	Behavior of health care seeking
He et al. (69)	China’s rural areas	766	SRH, MEI, FAI	Behavior of health care seeking
Zeng et al. (12)	China’s central and western regions	10,172	MEE, AGE, MEI, SRH	Behavior of health care seeking
Chen et al. (70)	Economically developed regions	831	TMI	Behavior of health care seeking
Dai et al. (71)	Beijing, China	2077	EDL, FAI, AHH, SRH	Behavior of health care seeking
Huang et al. (51)	China’s rural areas	2,647	MEI, SRH, EDL, AHH	Behavior of health care seeking
Du et al. (42)	Yanan, China	805	AHH, TMI	Behavior of health care seeking
Wang et al. (72)	Tangshan, China	6,171	EDL, MAS	Behavior of health care seeking
Qiu et al. (40)	Shanghai, China	397	ILS, SRH	Behavior of health care seeking
Xie et al. (73)	Fujian, China	568	EDL, MEI, FAI, DTM	Seek medical advice
Li et al. (74)	Xuzhou, China	763	FAI, DTM, ILS, AHH	Seek medical advice
Zhang et al. (32)	Weifang, China	154	MEE, MEI, DTM, AHH	Medical options
Du et al. (75)	Beijing, Shanghai, Shenzhen	3,034	MAS, EDL, FAI, MEI, ILS	Medical options
Jiang et al. (76)	Jiangsu, China	722	AGE, EDL, SRH, FAI, DTM	Medical options
Pu et al. (31)	Tianjin, China	235	FAI, MSR	Medical options
Pei et al. (77)	Central China region	1,312	TMI	Medical options
Li et al. (78)	China’s rural areas	1,102	SRH, MEE	Medical options
Su et al. (79)	China’s rural areas	769	MEE, DTM	Medical options
Duan et al. (80)	Beijing, China	781	EDL, FAI, MEE	Medical options
Cao et al. (81)	Gaoyou, China	1,384	AGE, EDL, FAI, ILS, MEI, MSR, DTM	Medical options
Zheng et al. (82)	China’s rural areas	3,156	AGE, EDL, FAI, MEI, SRH, ILS	Medical options
Shi et al. (83)	Chongqing, China	23,359	AGE, EDL, MAS, FAL, MEI, SRH, ILS, MEE	Choice of Medical Institutions
Li et al. (84)	Xuzhou, China	534	DTM, FAI, ILS, AHH	Medical options
Yin et al. (85)	Xuzhou, China	668	EDL, MAS, MEI, AHH, SRH	Medical options

AGE = Age, EDL = Education level, MAS = Marital Status, FAI=Family income, DTM = Distance to medical facilities, MEI = Medical Insurance, SRH=Self-rated health, HER = Health record, TMI = Trust in medical institution, MEE = Medical expenses, MSR = Healthcare service reputation, AHH = Awareness of hierarchical healthcare, ILS=Illness severity.

### 3.2 Data coding

In the coding process of meta-analysis, we focused on how BHS and the antecedent variables are measured instead of how they are labeled with the purpose to ensure the consistency among concept and definition of synonyms in different studies. With regard to the discrepancy issue, such as variables with similar meanings but different label, we reached a consensus through discussion.

After reaching a consensus on definitions and concepts of variables, we invite the first author and corresponding author to code the dependent and all of the antecedent variables independently at different time (with an interval of 1 month). We checked the consistency of two sets of coding results and reassessed the inconsistent results (20). This article uses observation queues and the Quality Assessment Tool for Observational Cohort and Cross Sectional Studies to evaluate the quality of literature. This evaluation tool consists of 14 items, each of which includes five options: yes, no, uncertain, unreported, and not applicable. The scoring standard is 1 point for “yes” and no points for the rest. The evaluation criteria for literature quality are good (total score >7), average (total score 5–7), and poor (total score <5). The two authors coded separately, with a consistency of Kappa = 0.813.

The results indicate that there are no significant differences in these two sets of coding results. We chose the correlation coefficient to capture the effect size. For articles that did not report the correlation coefficient, the t value, z value, and standardized regression coefficients are used to calculate the correlation coefficient.

### 3.3 The meta-analysis process

We followed Lipsey and Wilson (43) method to process the meta-analysis by using Comprehensive Meta Analysis 3.0 (CMA 3.0). Firstly, we conducted a descriptive analysis of correlation coefficients and calculated the effect sizes. In order to make the effect sizes exhibit normal distribution characteristics, Fischer’s Z-transform was used for the correlation coefficients. When the literature does not report the correlation coefficient, the correlation coefficient is calculated by *T*-value, *p*-value, etc.

Secondly, we adopted Q-test and $I^{2}$analysis to evaluate the heterogeneity of effect size. The results of the Q-test and $I^{2}$analysis are shown in Table 2. The results indicate that there is a significant heterogeneity among the studies.

**Table 2:** Heterogeneity test.

Variable relationship	*K*	Q value	df	*p* value	I-squared	Tau-squared
AGE-BHS	11	878.731	10	0.000	99.862	0.018
EDL-BHS	16	1934.8	15	0.000	99.225	0.033
MAS-BHS	8	1443.846	7	0.000	99.515	0.033
FAI-BHS	18	1988.169	17	0.000	99.145	0.034
DTM-BHS	11	345.867	10	0.000	97.109	0.011
MEI-BHS	15	964.965	14	0.000	98.549	0.020
SRH-BHS	18	4821.286	17	0.000	99.647	0.067
HER-BHS	4	397.555	3	0.000	99.245	0.034
TMI-BHS	8	1322.255	7	0.000	99.471	0.154
MEE-BHS	8	1435.247	7	0.000	99.512	0.075
MSR-BHS	9	6188.399	8	0.000	99.871	0.132
AHH-BHS	7	50.987	6	0.000	88.232	0.014
ILS-BHS	9	136.345	8	0.000	94.133	0.003

*K = number of research samples; df = degrees of freedom, Q vaule = Heterogeneity value, I-squared = The ratio of the variance between studies to the total variance, Tau Squared = between-group variance of the true effect. AGE = Age, EDL = Education level, MAS = Marital Status, FAI=Family income, DTM = Distance to medical facilities, MEI = Medical Insurance, SRH=Self-rated health, HER = Health record, TMI = Trust in medical institution, MEE = Medical expenses, MSR = Healthcare service reputation, AHH = Awareness of hierarchical healthcare, ILS=Illness severity.*

There are two meta-analysis models: fixed effects model and random effects model. The fixed effects model assumes that there is no heterogeneity (only sampling errors) between different studies, while the random effects model assumes the effect size is not fixed but heterogeneous. The random effects model is suggested to be adopted when there is high heterogeneity (44). According to the results of Q test and $I^{2}$analysis, this study chose the random effects model.

### 3.4 Publication bias

**Figure 3:**
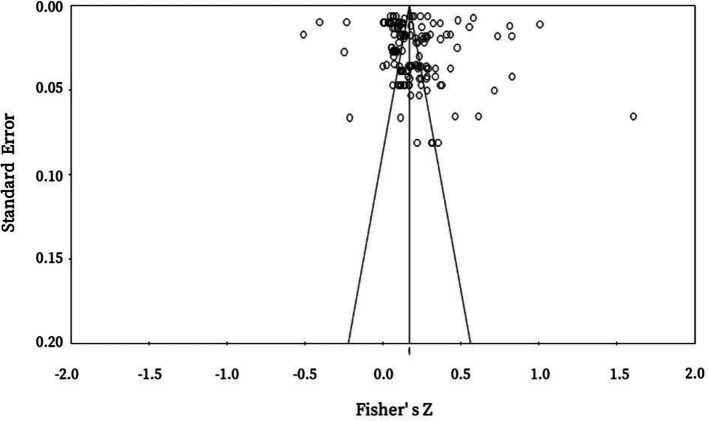
Funnel plot of publication deviation test.

According to Borenstein et al. (44), it may lead to overestimation of the true effects when meta-analysis focus on published studies and overlook ongoing or unpublished studies. Therefore, it is necessary to examine the distribution of effect size and evaluate the degree to which effect size deviates from the true value. In this study, we use the funnel plot test, Egger’s regression intercept, and Fail-safe N test to examine if there is a publication bias in our sample selection. The funnel plot shows that the plots do not take an inverted pyramid form (Figure 3). Therefore, it can be concluded that there is no significant bias issue. We also use the Egger’s regression intercept to test the publication bias (Table 3). The results of Egger’s regression interception showed that, except for the relationship between trust in medical institutions and BHS, as well as the relationship between awareness of hierarchical healthcare and BHS, the *p*-values of other variables were all greater than 0.05, indicating that the research results were not significant. Furthermore, the Fail-safe N test was conducted on the correlation of BHS with trust in medical institutions and awareness of hierarchical healthcare, and it was found that the safety factors of these correlations are greater than the critical value K × 5 + 10 (K is the number of literature), which indicates that the research results are reliable and there is no significant publication bias.

**Table 3:** Publication of bias analysis results.

Correlation variable	Egger’s regression intercept test			Fail-safe *N*
	95% confidence interval	*T*	*p*	
AGE-BHS	[−8.821, 15.720]	0.636	0.541	1,679
EDL-BHS	[0.352, 0.704]	0.388	0.70	7,881
MAS-BHS	[−17.322, 38.179]	0.920	0.393	2,805
FAI-BHS	[−2.071, 15.780]	1.628	0.123	8,559
DTM-HS	[−3.708, 8.969]	0.939	0.372	2,807
MEI-BHS	[−11.429, 5.974]	0.677	0.510	5,774
SRH-BHS	[−8.671, 24.928]	1.026	0.320	2,585
HER-BHS	[−59.337, 80.166]	0.642	0.586	383
TMI-BHS	[−35.487, −4.289]	3.120	0.02	2,259
MEE-BHS	[−17.722, 20.182]	0.159	0.880	330
MSR-BHS	[−59.288, 26.340]	0.910	0.393	4,362
AHH-BHS	[−24.897, −8.530]	4.380	0.000	4,563
ILS-BHS	[−7.136, 18.426]	1.135	0.308	233

### 3.5 P-curve analysis

**Figure 4:**
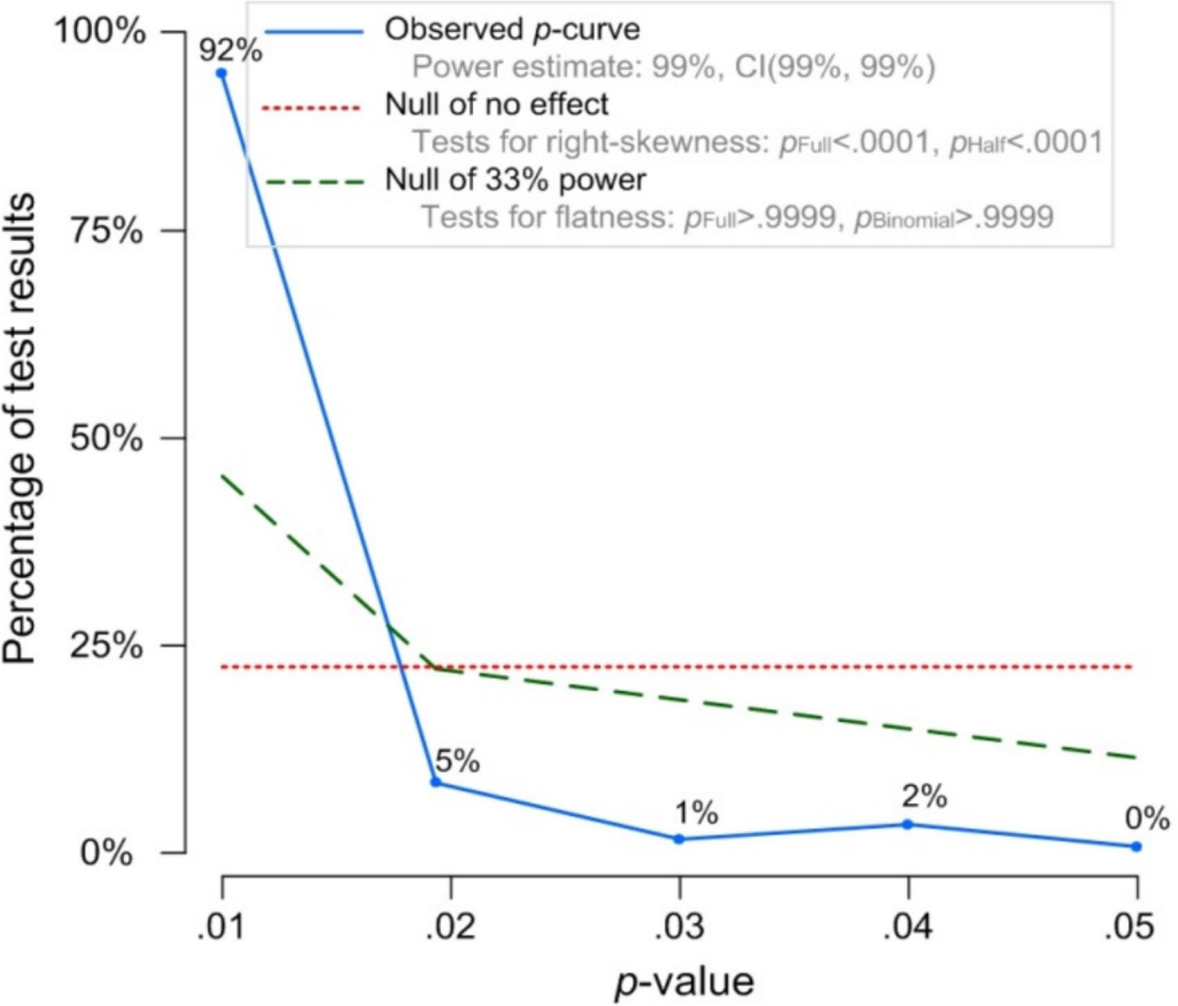
P-curve distribution. The observed p-curve includes 134 statistically significant (*p* < 0.05) results of which 130 are *p* < 0.025. There were 9 additional results entered but excluded from p-curve because they were *p* > 0.05.

The p-curve analysis was also applied to test the publication bias. If there is no significant publication bias, the *p*-value distribution should be skewed to the right, meaning that the number of *p*-values between 0 and 0.025 will exceed the number between 0.025 and 0.05 (45). According to the results of the p-curve analysis (Figure 4), the p-curve exhibits a right skewed distribution and out of 134 *p*-values, 130 have evidential value (*z* = 61.45), which indicates that the meta-analysis results are stable and reliable.

## 4 Results of the meta-analysis

### 4.1 Main effects

We analyzed the data for each hypothesis and first tested the main effects of antecedent variables on BHS (Table 4). According to the results, there is a significant and positive influence of the antecedent variables on BHS, with the exception of TMI (*z* = 0.176 and *p* = 0.077), HED (*z* = 1.942 and *p* = 0.052), and MEE (*z* = 0.1846 and *p* = 0.065). Furthermore, the relationship between FAI and BHS has the strongest weighted mean effect size (*r* = 0.272), while the relationship between AGE and BHS has the least weighted mean effect size (*r* = 0.1136). The reason for the insignificant impact of TMI, HER, and MEE on BHS may be that, on the one hand, the true effects between these variables are very small, and significant main effects may not be detected in meta-analysis. On the other hand, there are potential moderating variables that may influence the detection of the main effect.

**Table 4:** Results of random effects model analysis.

Variable relationship	Models	*K*	*r*	95%confidence interval		*Z*-value	*p*-value
				Lower limit	Upper limit		
AGE-BHS	random	11	0.136	0.057	0.214	3.340	0.001
EDL-BHS	random	16	0.205	0.118	0.289	4.550	0.000
MAS-BHS	random	8	0.185	0.061	0.304	2.904	0.004
FAI-BHS	random	18	0.272	0.190	0.350	6.348	0.000
DTM-BHS	random	11	0.230	0.166	0.292	6.889	0.000
MEI-BHS	random	15	0.175	0.103	0.245	4.723	0.000
SRH-BHS	random	18	0.147	0.028	0.262	2.420	0.016
HER-BHS	random	4	0.179	−0.002	0.347	1.942	0.052
TMI-BHS	random	8	0.242	−0.027	0.478	1.767	0.077
MEE-BHS	random	8	0.179	−0.011	0.357	1.846	0.065
MSR-BHS	random	9	0.253	0.020	0.460	2.128	0.033
AHH-BHS	random	7	0.198	0.105	0.287	4.134	0.000
ILS-BHS	random	9	0.155	0.120	0.191	8.459	0.000

*p < 0.05, **p < 0.01, ***p < 0.001; *k*= Number of research samples; *r* = Weighted average correlation coefficient corrected for sample size; *Z* = Z-value when the test is empty (2-tail).

### 4.2 Moderating effect

The results of the heterogeneity test show that there is a high degree of heterogeneity between the antecedent variables and BHS, which indicate that there might be a hidden moderator. Therefore, a moderation effect analysis is necessary to identify the contextual and methodological moderators responsible for heterogeneity in the effect size. According to the current status of Chinese healthcare system, medical resources allocation and medical service consumption in various regions largely depend on the regional economic development level. Therefore, this study will further explore the moderating effect of regional economic development level on the relationships between antecedent variables and patients’ BHS.

This article first identifies the regions where the 39 literature samples included in the analysis are located, including Beijing, Shanghai, Guangzhou, Tianjin, Jiangsu, Shaanxi, Ningxia, Fujian, and other regions. Secondly, according to the China Statistical Yearbook 2023, the above-mentioned regions are divided into developed areas (Beijing, Shanghai, Guangzhou, Tianjin, Jiangsu) and underdeveloped areas (Shaanxi, Ningxia, Fujian, etc.) based on their GDP. The results of the moderating effect of regional economic development level are presented in Table 5. The results indicate that there is no significant difference in the impact of marital status (MAS), distance to medical facilities (DTM), self-rated health (SRH), and awareness of hierarchical healthcare (AHH) on patients’ BHS in different regions based on their regional economic development level.

**Table 5:** The moderating role of a country’s level of economic development.

Variable relationship	Models	*K*	*r*	95%confidence interval		*Z*-value	QB
				Lower limit	Upper limit		
AGE-BHS	undeveloped	1	0.353	0.335	0.371	34.565	234.854^***^
	developed	3	0.212	0.157	0.233	3.023	
EDL-BHS	undeveloped	1	0.279	0.201	0.353	6.812	7.266^*^
	developed	8	0.261	0.068	0.436	2.626	
MAS-BHS	undeveloped	5	0.113	0.045	0.179	3.241	2.151
	developed	3	0.302	0.055	0.514	2.380	
FAI-BHS	undeveloped	1	0.326	0.250	0.398	8.042	28.009^***^
	developed	9	0.413	0.198	0.590	3.610	
DTM-BHS	undeveloped	3	0.402	−0.076	0.729	1.664	1.611
	developed	6	0.183	0.121	0.242	5.772	
MEI-BHS	undeveloped	2	0.175	0.103	0.245	4.723	39.577^***^
	developed	5	0.194	0.080	0.302	3.326	
SRH-BHS	undeveloped	2	0.221	0.031	0.396	2.268	4.236
	developed	6	0.271	0.166	0.370	4.922	
MSR-BHS	undeveloped	1	0.448	0.434	0.462	54.841	1114.078^***^
	developed	2	0.267	−0.093	0.566	1.642	
AHH-BHS	undeveloped	2	0.162	−0.125	0.424	1.110	0.147
	developed	3	0.200	0.118	0.279	4.733	
ILS-BHS	undeveloped	2	0.118	0.076	0.160	5.446	12.138^**^
	developed	4	0.228	0.163	0.290	6.704	

*p<0.05, **p<0.01, ***p<0.001; *K* = Number of research samples; *r* = Weighted average correlation coefficient corrected for sample size; *Z* = *Z*-value when the test is empty (2-tail); $Q_{B}$= Test statistic for heterogeneity between groups.

In addition, statistical support for moderated tests has the potential to mask the variability of existing studies, and unsupported information is masked out. The results of moderated tests may be affected by a variety of factors, including sample size, data distribution, and model setting (46). Bayesian ANOVA can more accurately portray the relationship between variables and make up for the shortcomings of moderated tests (47, 48). In addition, Bayesian ANOVA is more able to visualize the probability distribution of moderator effects relative to meta-regression techniques, thus enhancing the interpretability and credibility of the results (49). The results of the Bayesian analysis of variance are shown in Table 6. The results indicate that there is weak evidence that regional economic development level moderates the relationship between MAS and BHS, and there is weak evidence that the correlation of BHS with DTM, SRH, and MEE is not affected by the regional economic development level.

**Table 6:** Bayesian analysis.

	Models	*P* (M)	*P*(M|data)	*BF_M_*	*BF_10_*	% error
Variance analysis	Null model	0.500	0.428	0.748	1.000	0.006
	MAS-BHS (regions)	0.500	0.526	1.109	1.110	
	Null model	0.500	0.735	2.771	1.000	0.007
	DTM-BHS (regions)	0.500	0.469	0.882	0.882	
	Null model	0.500	0.620	1.634	1.000	0.009
	SRH-BHS (regions)	0.500	0.441	0.787	0.787	
	Null model	0.500	0.894	0.894	1.000	0.010
	AHH-BHS (regions)	0.500	0.528	1.119	1.120	

P (M) Represents the prior probability of the model before obtaining the observed data; P(M|data) denotes the posterior probability of the model after data; *BF_M_* denotes the change from P before posterior; *BF_10_* denotes the degree of support for *M_1_* in the observed data relative to *M_0_*; % error Indicates the error rate.

## 5 Discussion

### 5.1 Conclusions

This study comprehensively considers individual cognitive factors, demographic factors, and medical level factors based on ABMHSU. We adopted the meta-analysis method and conducted a statistical analysis on 39 empirical articles, and finally verified the influence of individual cognitive factors (TMI, SRH, DTM, ILS, MEE), demographic factors (AGE, FAI, EDL, MAS), and medical level factors (MEI, AHH, HER, MSR) on BHS. In addition, this article examined the moderating effect of the regional economic development level on the relationship between antecedent variables and BHS.

In Demographic factors, there is a moderate positive correlation (*r* = 0.136, 0.205, 0.185, 0.272) between Age (H1), Education level (H2), Family income (H3), Marital status (H4), and patients’ BHS. Among the Individual cognitive factors, there was a low-strength positive correlation (*r* = 0.230, 0.175) between trust in healthcare providers (H5), healthcare spending (H6), and patients’ BHS; a medium-strength positive correlation (*r* = 0.147) between distance to healthcare (H7) and patients’ BHS; and there was no significant correlation between self-assessment of health (H8) and severity of illness (H9) and patients’ BHS (*p* = 0.052, 0.077). Among the Medical-level factors, there was no significant correlation between health insurance coverage (H10) and patients’ BHS (*p* = 0.065); there was a medium-strength positive correlation between healthcare facility reputation (H11) and health records (H12), and perceptions of hierarchical diagnosis and treatment (H13) and patients’ BHS (*r* = 0.253, 0.198, 0.155). Furthermore, the relationship between FAI and BHS has the strongest weighted mean effect size (*r* = 0.272), while the relationship between AGE and BHS has the least weighted mean effect size (*r* = 0.136).

Secondly, there is no significant difference in the impact of MAS, DTM, SRH, and AHH on patients’ BHS in terms of regional economic development level. The results show that in economically developed regions, the impact of FAI, MEI, and ILS on patients’ BHS is stronger than in economically underdeveloped regions, whereas the impact of AGE, EDL, and MSR on patients’ BHS is stronger in underdeveloped regions than in developed regions.

Finally, the results of the Bayesian analysis of variance show that there is weak evidence that the regional economic development level can significantly moderate the influence of MAS on patients’ BHS, and there is weak evidence that the moderating effect of regional economic development level on the relationship between DTM, SRH, AHH and BHS is insignificant.

### 5.2 Theoretical implications

This study has made certain theoretical contributions to the design of healthcare service systems, especially in the research on patients’ BHS in developing countries. To our knowledge, this study is one of the first to investigate the influencing factors of Chinese patients’ BHS using meta-analysis. By integrating relevant research on the influencing factors of Chinese patients’ BHS, this study summarizes the factors that affect patients’ BHS into three categories: demographic characteristics, individual cognition, and medical system. On the one hand, this study enhances the systematic and holistic research on the influencing factors of patients’ BHS, and once again confirms the impact of each influencing factor on patients’ BHS. On the other hand, we categorize the medical-level factors as environmental factors in ABMHSU, and empirically verify the impact of medical-level factors on personal hygiene behavior and healthcare service utilization in ABMHSU, which enhance the applicability of ABMHSU in Chinese context.

The correlation between AGE and medical care is relatively weak. On the one hand, young adults between the ages of 20 and 40 in contemporary China are in the ascending stage of their professional careers, and they face tremendous work pressure and heavy family responsibilities, which makes it difficult for them to find the time and energy to healthcare seeking. On the other hand, young people generally have a “delay medical treatment if symptoms are tolerable” mentality, and view medical services as a non-essential consumer goods.

The insignificant impact of TMI may be explained by the situation of Chinese medical system. In China, the extremely unreasonable compensation mechanism of public hospitals and the commercial interests mixed in medical practices have led to a generally low level of patients’ trust in medical institutions/doctors. In addition, healthcare service has the features of credence goods (11). Therefore, in China, patients’ trust in medical institutions/doctors mainly manifests as acquaintance trust in human society (50), rather than competence trust, goodwill trust, and institutional trust in existing studies. This conclusion can further promote the conceptualization and operationalization of doctor-patient trust in the Chinese context. Regarding MEE, on the one hand, medical insurance, which covers over 95% of the Chinese population, bears the majority of patients’ medical expenses. On the other hand, there is not much difference in the reimbursement ratio of medical insurance among different healthcare institutions, which makes the impact of MEE on BHS not significant. With regard to HER, the current medical system in China still focuses on “disease treatment,” and has not established an integrated medical and health system of “health prevention + disease treatment.” On the one hand, healthcare institutions lack the motivation to provide integrated healthcare services of “prevention and treatment” through the establishment of heath record and the ability of “preventive treatment.” On the other hand, patients are lack of health awareness of early detection and treatment of diseases through health monitoring. Therefore, the role of HER in Chinese patients’ BHS is not significant.

The heterogeneity analysis results show that the relationship between various antecedent variables and BHS is influenced by regional economic development level. However, this study did not find significant differences in the influence of DTM and SRH on BHS at different regional economic development levels. This is because with the improvement of public health awareness, even if there are differences in regional economic development level and the resulting differences in medical resource allocation, patients will still try their best to choose suitable healthcare institutions for treatment, and will not compromise or even refuse to seek healthcare due to a lack of high-quality medical resources. This also explains to some extent the current situation of patients from all over China traveling thousands of miles to cities (such as Beijing and Shanghai) with abundant high-quality medical resources for treatment. Secondly, in the context of China’s vigorous promotion of hierarchical healthcare and full coverage of basic medical insurance, although the implementation effect of hierarchical healthcare varies slightly in different regions, there is no difference in the promotion of hierarchical healthcare policies, which may lead to no significant difference in the impact of AHH on BHS at different regional economic development level. In addition, the moderating effect of regional economic development level reveals systematic contradictions in the distribution of healthcare resources. For example, FAI and MEI are stronger in economically developed regions, while AGE and EDL dominate more significantly in less developed regions. The co-existence of “supply-side shortages” (e.g., uneven distribution of quality healthcare resources) and “demand-side barriers” (e.g., low health literacy among rural residents) requires policymakers to adopt a differentiated strategy: optimizing the allocation of resources through hierarchical diagnosis and treatment in urban areas, and strengthening the construction of primary healthcare facilities and the promotion of health science and technology in rural areas.

Finally, although this study did not find significant difference in the influence of MAS on BHS across different regional economic development levels, the results of Bayesian analysis shows weak evidence that regional economic development levels can significantly moderate the influence of MAS on BHS. This may be because although there are no significant differences in marriage attitudes among different regions under the influence of traditional Chinese culture, different customs and traditions may have a subtle impact on the level of support of marital status for the care of patients.

### 5.3 Managerial implications

For regulatory authorities, in order to guide patients to seek healthcare reasonably, maximize the value of medical resources and improve the efficiency of the hierarchical healthcare system, on the one hand, they should increase the sinking of high-quality healthcare resources and optimize the mechanism of two-way referral through medical consortia. Meanwhile, they should increase the support for primary care in terms of medical insurance payment scope and payment ratio with the purpose to guide patients to enter the healthcare service system through primary healthcare institutions. On the other, they should collaborate with healthcare institutions to increase their publicity efforts on the policy of hierarchical healthcare, healthcare knowledge, etc., and eventually improve public trust in the healthcare system. For example, the regulatory authorities should disseminate the discrimination between self-limiting diseases and self-healing disease to the public, guide patients to pursue appropriate medical intervention according to the progress of self-limiting disease, and recommend patients to reduce seeking medical intervention regarding self-healing disease with the purpose to minimize the excessive medical treatment and strengthen the immune competence of patients.

For healthcare institutions, they should improve the quality of healthcare services and patient experience by innovating healthcare service models, optimizing healthcare service processes, and improving healthcare technology capabilities, and eventually improving the healthcare service reputation and gaining the competence trust and goodwill trust of patients. For example, primary healthcare institutions can provide health monitoring and other services to residents in their jurisdiction through establishing health record, and simplify the upward referral process within the medical consortia aiming to improve the accessibility of high-quality medical services.

For patients, in order to improve the effectiveness of healthcare services, it is need to provide doctors with information such as symptoms, disease perception, and medical history to the greatest extent based on balancing personal privacy and data sharing. Patients should shift the role from “passively receiving treatment” to “actively participating in the medical service process.” In addition, patients should cooperate with regulatory authorities and healthcare institutions to strengthen their learning of healthcare knowledge. Accordingly, patients can assess their own health status more accurately, treat the healthcare service results more rationally and reduce doctor shopping (51), and eventually reduce congestion in the healthcare system.

### 5.4 Limitations and future research

We recognize the following limitations. Firstly, we have searched literatures regarded to influencing factors of BHS as far as possible with the purpose to minimize the selection bias, however we only searched the online full-text articles published in English and Chinese. Although the publication bias analysis did not find significant publication bias, it may still have some impact on the research results. As regards methodology, due to the limitations of meta-analysis methods, we excluded literature that did not disclose basic information such as correlation coefficients and non-empirical studies, which may overlook some important factors that affect patients’ BHS. Therefore, future research should integrate qualitative analysis articles through qualitative meta-analysis (52). On one hand, as doing so can reduce literature selection bias; on the other hand, it can minimize the p-hacking problem in empirical studies to the greatest extent (53).

Secondly, this study only examined on the direct relationship between antecedent variables and BHS, without considering the interrelationships between various antecedent variables and potential mediating effects. It is necessary for future research to use meta-analysis structural equation modeling to identify the complex interaction relationships between antecedent variables and potential mediating effects (21).

Finally, due to the limitations in sample information, this study only analyzed the moderating effect of regional economic development level, without considering other potential moderators. Future research can analyze the influence of many other moderators, such as disease types (chronic vs. acute), doctor-patient relationships, etc., or consider the influence of healthcare policies, medical insurance policies, and culture beyond national limitations.

## Data Availability

The original contributions presented in the study are included in the article/supplementary material, further inquiries can be directed to the corresponding author.

## References

[1] Godager G Iversen T Ma CA. Competition, gatekeeping, and health care access. J Health Econ. (2015) 39:159–70. doi: 10.1016/j.jhealeco.2014.11.005, PMID: 25544400

[2] Mwabu GM. Referral systems and health care seeking behavior of patients: an economic analysis. World Dev. (1989) 17:85–91. doi: 10.1016/0305-750X(89)90224-6

[3] Freeman M Savva N Scholtes S. Gatekeepers at work: an empirical analysis of a maternity unit. Manag Sci. (2016) 63:3147–67. doi: 10.1287/mnsc.2016.2512, PMID: 19642375

[4] Zhou Z Zhao Y Shen C Lai S Nawaz R Gao J. Evaluating the effect of hierarchical medical system on health-seeking behavior: a difference-in-differences analysis in China. Soc Sci Med. (2021) 268:113372. doi: 10.1016/j.socscimed.2020.113372, PMID: 32979776

[5] Li X Krumholz HM Yip W Cheng K DeMaeseneer J Meng Q. Quality of primary health care in China: challenges and recommendations. Lancet. (2020) 395:1802–12. doi: 10.1016/S0140-6736(20)30122-7, PMID: 32505251 PMC7272159

[6] Li Z. Government subsidy schemes for coordinating healthcare referral in hierarchical service systems. J Indus Eng Manage. (2023) 27:103–17.

[7] Cheng MC Liu SP Chuang YC Hsu KCP Chow PM. Prevalence and impacts of male urinary incontinence on quality of life, mental health, work limitation, and health care seeking in China, Taiwan, and South Korea (LUTS Asia): results from a cross-sectional, population-based study. Invest Clinic Urology. (2021) 63:71–82. doi: 10.4111/icu.20210259, PMID: 34983125 PMC8756147

[8] Handebo S Adugna A Kassie A Wolde M Shitu K. Health care seeking behavior for common childhood illnesses in Ethiopia: a systematic review and meta-analysis. J Public Health. (2023) 31:1533–45. doi: 10.1007/s10389-022-01692-5

[9] Nab M Van Vehmendahl R Somers I Schoon Y Hesselink G. Delayed emergency healthcare seeking behavior by Dutch emergency department visitors during the first COVID-19 wave: a mixed methods retrospective observational study. BMC Emerg Med. (2021) 21:56. doi: 10.1186/s12873-021-00449-9, PMID: 33932988 PMC8087882

[10] Santos R Gravelle H Propper C. Does quality affect patients’ choice of doctor? Evidence from England. Econ J. (2017) 127:445–94. doi: 10.1111/ecoj.12282, PMID: 28356602 PMC5349292

[11] Gottschalk F Mimra W Waibel C. Health services as credence goods: a field experiment. Econ J. (2020) 130:1346–83. doi: 10.1093/ej/ueaa024, PMID: 29053835

[12] Zeng Y Wan Y Yuan Z Fang Y. Healthcare-seeking behavior among Chinese older adults: patterns and predictive factors. Int J Environ Res Public Health. (2021) 18:2969. doi: 10.3390/ijerph18062969, PMID: 33799366 PMC7998758

[13] Kombate G Cakpo GE Azianu KA Labité MA van der Sande MA. Care-seeking behaviour among febrile children under five in Togo. BMC Public Health. (2022) 22:2107. doi: 10.1186/s12889-022-14550-6, PMID: 36397027 PMC9670432

[14] Murphy M Scott LJ Salisbury C Murphy M Scott LJ Salisbury C . Implementation of remote consulting in UK primary care following the COVID-19 pandemic: a mixed-methods longitudinal study. Br J Gen Pract. (2021) 71:e166–77. doi: 10.3399/BJGP.2020.0948, PMID: 33558332 PMC7909923

[15] Zeng Y Xu W Chen L Chen F Fang Y. The influencing factors of health-seeking preference and community health service utilization among patients in primary care reform in Xiamen China. Patient Prefer Adher. (2020) 14:653–62. doi: 10.2147/PPA.S242141, PMID: 32280202 PMC7125321

[16] Tesema GA Seifu BL. Factors associated with mother’s healthcare-seeking behavior for symptoms of acute respiratory infection in under-five children in sub-Saharan Africa: a multilevel robust Poisson regression modeling. BMC Health Serv Res. (2023) 23:1061. doi: 10.1186/s12913-023-10065-x, PMID: 37794438 PMC10552283

[17] Osman AF Mutalib MA Tafran K Tumin M Chong CS. Demographic and socioeconomic variables associated with health care–seeking behavior among foreign workers in Malaysia. Asia-Pac J Public He. (2020) 32:42–8. doi: 10.1177/1010539519893801, PMID: 31924113

[18] Yadav S Choi T Kumar A Luthra S Naz F. A meta-analysis of sustainable supply chain practices and performance: the moderating roles of type of economy and innovation. Int J Oper Prod Man. (2023) 43:802–45. doi: 10.1108/IJOPM-05-2022-0328

[19] Iftikhar A Purvis L Giannoccaro I. A meta-analytical review of antecedents and outcomes of firm resilience. J Bus Res. (2021) 135:408–25. doi: 10.1016/j.jbusres.2021.06.048

[20] Chen L Jia F Li T Zhang T. Supply chain leadership and firm performance: a meta-analysis. Int J Prod Econ. (2021) 235:108082. doi: 10.1016/j.ijpe.2021.108082

[21] Oduro S De Nisco A Mainolfi G. Do digital technologies pay off? A meta-analytic review of the digital technologies/firm performance nexus. Technovation. (2023) 128:102836. doi: 10.1016/j.technovation.2023.102836

[22] Lemming MR Calsyn RJ. Utility of the Behavioral model in predicting service utilization by individuals suffering from severe mental illness and homelessness. Community Ment Hlt J. (2004) 40:347–64. doi: 10.1023/B:COMH.0000035229.20557.5c, PMID: 15453086

[23] Lo TKT Parkinson L Cunich M Byles J. Factors associated with the health care cost in older Australian women with arthritis: an application of the Andersen’s behavioural model of health services use. Public Health. (2016) 134:64–71. doi: 10.1016/j.puhe.2015.11.018, PMID: 26791096

[24] Thompson AE Anisimowicz Y Miedema B Hogg W Wodchis WP Aubrey-Bassler K. The influence of gender and other patient characteristics on health care-seeking behavior: a QUALICOPC study. BMC Fam Pract. (2016) 17:1–7. doi: 10.1186/s12875-016-0440-0, PMID: 27036116 PMC4815064

[25] Thomas BE Thiruvengadam K Raghavi S Rani S Vetrivel S Rao VG . Understanding health care-seeking behavior of the tribal population in India among those with presumptive TB symptoms. PLoS One. (2021) 16:e0250971. doi: 10.1371/journal.pone.0250971, PMID: 34014938 PMC8136700

[26] Gamtesa DF Tola HH Mehamed Z Eesfaye E Alemu A. Health care seeking behavior among presumptive tuberculosis patients in Ethiopia: a systematic review and meta-analysis. BMC Health Serv Res. (2020) 20:445–10. doi: 10.1186/s12913-020-05284-5, PMID: 32429988 PMC7238571

[27] Kundu S Nizum MWR Fayeza F Chowdhury SSA Bakchi J Sharif AB. Magnitude and trends in inequalities in healthcare-seeking behavior for pneumonia and mortality rate among under-five children in Bangladesh: evidence from nationwide cross-sectional survey 2007 to 2017. Health Sci Rep. (2013) 6:e1744. doi: 10.1002/hsr2.1744, PMID: 38078306 PMC10700677

[28] Zewude B Siraw G Engdawork K Tadele G. Health seeking behavior of street connected children in Addis Ababa, Ethiopia. Front Sociol. (2023) 8:1188746. doi: 10.3389/fsoc.2023.1188746, PMID: 37609109 PMC10441109

[29] Tekalign T Guta MT Awoke N Asres AW Obsa MS. Mothers’ care-seeking behavior for common childhood illnesses and its predictors in Ethiopia: meta-analysis. Int Journal of Pediatr. (2022) 2022:2221618. doi: 10.1155/2022/2221618, PMID: 36304521 PMC9596259

[30] Nagdev P Iyer MR Naik S Khanagar B Awawdeh M Kheraif A . Andersen health care utilization model: a survey on factors affecting the utilization of dental health services among school children. PLoS One. (2023) 18:e0286945. doi: 10.1371/journal.pone.0286945, PMID: 37319189 PMC10270576

[31] Pu HJ Liu XT Yu KY Wang LX Zhang TW Zhang Y. Study on choice of medical treatment of elderly people: based on binary logistic regression model. World Chongqing. (2015) 11

[32] Zhang CP Bing LF Yue LL Xing J Guo JM. Analysis on the choice and influencing factors of Residents’ first visit under the hierarchical diagnosis and treatment system——taking Weifang as an example. China Health Insur. (2020) 2:57–63.

[33] Sivey P. The effect of waiting time and distance on hospital choice for English cataract patients. Health Econ. (2012) 21:444–56. doi: 10.1002/hec.1720, PMID: 21384464

[34] Akter S Banna MHA Brazendale K Sultana MS Kundu S Disu TR . Determinants of health care seeking behavior for childhood infectious diseases and malnutrition: a slum-based survey from Bangladesh. J Child Health Care. (2023) 27:395–409. doi: 10.1177/13674935211057714, PMID: 35164525

[35] Agarwal P Wang R Meaney C Walji S Damji A Gill N . Sociodemographic differences in patient experience with virtual care during COVID-19: results from a cross-sectional survey in Ontario, Canada. BMJ Open. (2022) 12:e056868. doi: 10.1136/bmjopen-2021-056868, PMID: 35534055 PMC9086266

[36] Jin Y Yuan B Zhu W Zhang Y Xu L Meng Q. The interaction effect of health insurance reimbursement and health workforce on healthcare-seeking behavior in China. Int J Health Plan Manage. (2019) 34:900–11. doi: 10.1002/hpm.2860, PMID: 31353637

[37] Cao N Li X Jiang J Xu W. The effect of basic medical insurance on the changes of primary care seeking behavior: an application of hierarchical age-period-cohort analysis. Front Public Health. (2022) 10:929896. doi: 10.3389/fpubh.2022.929896, PMID: 35991071 PMC9382404

[38] Wu Y Wang Q Zheng F Yu T Wang Y Fan S . Effects of the implementation of transport-driven poverty alleviation policy on health care–seeking behavior and medical expenditure among older people in rural areas: quasi-experimental study. JMIR Public Hlth Sur. (2023) 9:e49603 10.2196/49603 PMC10716743 38015603

[39] Wang Y Wu H Lei XQ Shen JX Feng ZC. The influence of doctors’ online reputation on the sharing of outpatient experiences: empirical study. J Med Internet Res. (2020) 22:e16691. doi: 10.2196/16691, PMID: 33306028 PMC7762689

[40] Qiu C Zhang Y Wang X Gu D. Trust-based research: influencing factors of patients’ medical choice behavior in the online medical community. Healthcare. (2022) 10:938. doi: 10.3390/healthcare10050938, PMID: 35628075 PMC9140699

[41] Lai S Gao JM Yang XW Li Q. Studying on the health-seeking behaviors of rural patients with chronic non-communicable diseases under the background of new medical reform based on household health interview survey in rural Shaanxi. Chin Health Ser Manage. (2015)

[42] Du T Liu ZZ Yang JL. Research on the patients’ choice behavior of preferred medical institutions in the regional medical association. Health Econ Res. (2022) 39:40–4.

[43] Lipsey MW Wilson DB. Practical meta-analysis SAGE publications, Inc. (2001).

[44] Borenstein M Hedges LV Higgins JPT Rothstein HR. A basic introduction to fixed-effect and random-effects models for meta-analysis. Res Synth Methods. (2010) 1:97–111. doi: 10.1002/jrsm.12, PMID: 26061376

[45] Farajzadeh A Hébert A Lahart IM Bilodeau M Boisgontier MP. Apathy and physicalactivity: a systematic review and meta-analysis. Med Rxiv. (2024):2024–05. doi: 10.1101/2024.05.01.24306712

[46] Jadil Y Rana NP Dwivedi YK. A meta-analysis of the UTAUT model in the mobile banking literature: the moderating role of sample size and culture. J Bus Res. (2021) 132:354–72. doi: 10.1016/j.jbusres.2021.04.052

[47] Song YT Wu MZ. Quality management and firm innovation: linking Bayesian network and structural equations model. Modern Manage. (2024) 44:120–30. doi: 10.19634/j.cnki.11-1403/c.2024.06.012

[48] Cumming G. The new statistics: why and how. Psychol Sci. (2014) 25:7–29. doi: 10.1177/0956797613504966, PMID: 24220629

[49] Kruschke JK Liddell TM. The Bayesian new statistics: hypothesis testing, estimation, meta-analysis, and power analysis from a Bayesian perspective. Psychon Bull Rev. (2018) 25:178–206. doi: 10.3758/s13423-016-1221-4, PMID: 28176294

[50] Li X Sun X Shao Q. Trust in Acquaintances, strangers and institutions among individuals of different socioeconomic statuses during public health emergencies: the moderation of family structure and policy perception. Behav Sci. (2024) 14:404. doi: 10.3390/bs14050404, PMID: 38785894 PMC11118019

[51] Huang F Guo P Wang Y. Modeling patients’ illness perception and equilibrium analysisof their doctor shopping behavior. Prod Oper Manag. (2022) 31:1216–34. doi: 10.1111/poms.13606

[52] Combs JG Crook TR Rauch A. Meta-analytic research in management: contemporary approaches, unresolved controversies, and rising standards. J Manage Stud. (2018) 56:1–18. doi: 10.1111/joms.12427, PMID: 40189918

[53] Brinkerink J. When shooting for the stars becomes aiming for asterisks: P-hacking in family business research. Entrep Theory Pract. (2023) 47:304–43. doi: 10.1177/10422587211050354

[54] Liu Q Hu SX Yang BW. Research on the influence of residents’ cognitive level of diagnosis and treatment on medical treatment behavior. Chin Hospital Manage. (2022) 42:20–4.

[55] Chen C Zheng M Gao YR Lu W Zhu JL. Study on the influencing factors of residents’ healthcare seeking behavior in China under the background of hierarchical medical system—based on CHNS data. Chin Hospitals. (2022) 26:19–22. doi: 10.19660/j.issn.1671-0592.2022.6.06

[56] Wang M Zhang KK Jiang L Huang X Bao SM. Impact factors model of medical behavior of Chinese urban and rural ill residents. Chin General Prac. (2010) 13:2127–9.

[57] Zhang XX Qin C Yang X Chen L. Influencing factors on medical treatment choice and hospitalization expenses under the background of real-time reimbursement policy for medical treatment in non-resident place: an empirical study of City a. Med Soc. (2021) 34:54–8. doi: 10.13723/j.yxysh.2021.06.011

[58] Song QC Yin K. Study on the first diagnosis choice and its influencing factors of the elderly migrants in China. Dong Yue Tribune. (2021) 42:136-147+192. doi: 10.15981/j.cnki.dongyueLuncong.2021.01.014

[59] Zeng YY Guo DM. Patients’ behavior of selecting physicians in online health community based on trust perspective: taking the website of www. Haodf. Com as an example. Inform Stud. (2018) 41:96-101+113.

[60] Wu XL Zhang ZZ. Study on the online psychological consultation users’ preferences for Dotor selection. J Med Inform. (2021) 42:22-27+33.

[61] Yi MX Wu J Cai JX Gao JH. Online doctor selection behavior based on multi-source information of text and pictures from the perspective of trust. Inform Sci. (2021) 39:84–93. doi: 10.13833/j.issn.1007-7634.2021.09.012

[62] Wei J Yang ZL. Influence of patient-generated, doctor-generated, and system generated content on patients′ behavior of selecting doctors. J. Manage Sci. (2022) 35:44–56. doi: 10.3969/j.issn.1672-0334.2022.04.004

[63] Wu JH Liu Q Tao L. Selection of a physician by patients in online healthcare community: an elaboration likelihood model perspective. J Guangdong Univ Technol. (2022) 39:8–15.

[64] Li DD Guo W Guo YB Lin Z Wu P Wang Y. The impact of the distance between the place of residence and the nearest medical institution on residents’ medical behavior. Chin J Health Statis. (2020) 37:269-271+275.

[65] Feng X Hua ZL Zhou Q Shi AW Qian DF. Study on the health seeking behavior and influencing factors of middle-aged and elderly residents in a City from the perspective of social support network. Chin Health Serv Manage. (2020) 37:317–20.

[66] He AQ Yu Y Zheng S Liang J. Influencing factors of medication seeking behaviors among adult migrant population with chronic diseases: a hierarchical model-based analysis. Chin J Public Health. (2022) 38:75–9. doi: 10.11847/zgggws1127307

[67] Li ZL Ma C. Research on the behavior of migrant workers participating in urban employee medical insurance and medical treatment in Jiangsu Province. Chin J Health Statis. (2021) 38:746–9. doi: 10.3969/j.issn.1002-3674.2021.05.028

[68] He D Fan SS Du FY. Research on the influencing factors of children’s medical behavior in Beijing: a case study of Beijing Children’s hospital and Capital Institute of Pediatrics. J Beijing Union Univ. (2022) 36:44–52. doi: 10.16255/j.cnki.ldxbz.2022.03.008

[69] He YT Yu BT Wang RN Zhang YL. Influencing factors of rural women’s healthcare-seeking choice behavior based on China family panel studies data in 2018. Modern Preven Med. (2022) 49:4457–61. doi: 10.20043/j.cnki.MPM.202203622

[70] Chen SQ Guo XT Wu TS Ju XF. Exploring the influence of doctor–patient social ties and knowledge ties on patient selection. Internet Res. (2021) 32:219–40. doi: 10.1108/INTR-07-2020-0403, PMID: 35579975

[71] Dai YH Wang T Yu HX Bu XX. Status and associated factors of Patients’ Behaviors of seeking community-based outpatient services after the comprehensive medical reform of separating drug sales from medical treatment launched in Beijing. Chin General Prac. (2019) 22:24-31.

[72] Wang SS Hao XJ Li X Chen CX. The impact of cardiovascular and cerebrovascular diseases on the quality of life and medical behavior of elderly people. Chin J Gerontol. (2022) 42:719–21. doi: 10.3969/j.issn.1005-9202.2022.03.057

[73] Xie XY Zhao XJ Wu QD Wu Y Wei Q. Analysis of influencing factors on the choice of medical treatment among the public within the Fujian medical association. Chin J Health Statis. (2022) 39:755–9. doi: 10.3969/j.issn.1002-3674.2022.05.027

[74] Li HH Miao CX Zhuo L Jiang JX Wang WH Zheng J . Choices of medical treatment analysis based on hierarchical diagnosis and treatment system among rural residents, Xuzhou. Modern Preven Med. (2017) 44:2768-2771+2783.

[75] Du BF Miao F. Medical orientation of young migrants: evidence from Beijing, Shanghai and Shenzhen Cities. Population Res. (2012) 36:71–86.

[76] Jiang HJ Yao ZY. Influence factor analysis of medical choice and medical expense for wowen in rural areas of Jiangsu Province. Jiangsu J Prev Med. (2015) 26:30–2. doi: 10.13668/j.issn.1006-9070.2015.04.011

[77] Pei Y Wang ZW Che YH Fan J Liu XJ Niu F. Analysis of the influence of the credit rating of medical institutions on patients’ choices of medical service. Chin Hospital Manage. (2018) 38:50–2.

[78] Li WL Zhou SY Yu Y. Medical and health security, allocation of health resources, and rural labor’s choice of medical treatment: based on the analysis of 2016 CLDS data. Soc Sci Hunan. (2019) 1:81–7.

[79] Su J Gao X Lei XY Hu HP. The influencing factors of health-seeking behaviors of patients with chronic diseases. China Med Herald. (2020) 17:174–7. doi: 10.20047/j.issn1673-7210.2020.14.042

[80] Duan H Liu Y Zhang YN Liu C. Study on the influencing factors of the medical treatment choices of chronic disease patients under the background of Beijing integrated health care system. Chin J Health Policy. (2020) 13:14–20.

[81] Cao Y Cheng TS. Study on the influencing factors of medical treatment choice of urban chronic disease patients in Gaoyou City. Med Soc. (2020) 33:37–43. doi: 10.13723/j.yxysh.2020.10.008

[82] Zheng YH Hao XN. Research on medical orientation and influence factors of elderly floating population. Chin Health Econ. (2021) 40:56–9.

[83] Shi SJ Miao CX Huang C Yin YN Sun H Huang XJ . Studying on the residents’ choice of medical institutions in China and its influencing factors based on Shapley value method. Chin Health Serv Manage. (2022) 39:509-512+526.

[84] Li HY Jiang JX Zhao QY Jiang J. Associated factors for choice of medical institutions for treatment in chronic disease patients during the implementation of hierarchical medical system: a survey in Xuzhou. Chin General Prac. (2020) 23:1546–51.

[85] Yin YN Miao CX Huang XJ Li B Fang X Gao XY. Study on choice of medical treatment of floating population in Xuzhou based on Andersen model. Med Soc. (2023) 36:58–63. doi: 10.13723/j.yxysh.2023.02.011

